# Validation study of MARCKSL1 as a prognostic factor in lymph node-negative breast cancer patients

**DOI:** 10.1371/journal.pone.0212527

**Published:** 2019-03-11

**Authors:** Nina Gran Egeland, Marie Austdal, Bianca van Diermen-Hidle, Emma Rewcastle, Einar G. Gudlaugsson, Jan P. A. Baak, Ivar Skaland, Emiel A. M. Janssen, Kristin Jonsdottir

**Affiliations:** 1 Department of Pathology, Stavanger University Hospital, Stavanger, Norway; 2 Department of Chemistry, Bioscience and Environmental Engineering, University of Stavanger, Stavanger, Norway; 3 Department of Breast and Endocrine Surgery, Stavanger University Hospital, Stavanger, Norway; 4 Dr. Med. Jan Baak AS, Tananger, Norway; University of South Alabama Mitchell Cancer Institute, UNITED STATES

## Abstract

Protein expression of Myristoylated alanine-rich C kinase substrate like-1 (MARCKSL1) has been identified as a prognostic factor in lymph-node negative (LN^-^) breast cancer patients. We aim to validate MARCKSL1 protein expression as a prognostic marker for distant metastasis-free survival (DMFS) in a new cohort of LN^-^ breast cancer patients. MARCKSL1 expression was evaluated in 151 operable T_1,2_N_0_M_0_ LN^-^ breast cancer patients by immunohistochemistry. Median follow-up time was 152 months, range 11–189 months. Results were compared with classical prognosticators (age, tumor diameter, grade, estrogen receptor, and proliferation) using single (Kaplan-Meier) and multivariate (Cox model) survival analysis. Thirteen patients (9%) developed distant metastases. With both single and multiple analysis of all features, MARCKSL1 did not show a significant prognostic value for DMFS (p = 0.498). Of the assessed classical prognosticators, only tumor diameter showed prognostic value (hazard ratio 9.3, 95% confidence interval 2.8–31.0, p <0.001). MARCKSL1 expression could not be confirmed as a prognostic factor in this cohort. Possible reasons include changes in diagnostic and treatment guidelines between the discovery and validation cohorts. Further studies are needed to reveal the potential biological role of this protein in breast cancer.

## Introduction

Breast cancer is a leading cause of death for women in the western world. In Norway women have a cumulative risk of 8.6% for developing the disease before the age of 75 [1]. About 50% of these women will present with stage I-II lymph-node negative (LN^-^) cancer [2]. Stage I-II patients generally have a good prognosis, with a five-year survival rate of 89–99% [1], and not all may benefit from additional chemotherapy [3]. Still, approximately 12% of LN^-^ patients will experience recurrence, even up to 20 years after the initial diagnosis [4–6]. This, in combination with few recurrences among many patients with a good prognosis, leads to both over- and undertreatment of patients. Consequently, there is a need for new prognostic factors, as unnecessary chemotherapy and radiation may cause late-onset adverse effects, which reduce the quality of life of cancer survivors [7, 8].

Previous research has identified several proliferation markers such as phosphohistone H3 (PPH3), Ki-67 and mitotic activity index (MAI) as prognostic factors for LN^-^ breast cancer [9–13]. PPH3 demarks mitotic chromatin condensation, a late stage in mitosis, while Ki-67 is a nuclear protein that is expressed in all active phases of the cell cycle [10]. MAI, which is the number of cells undergoing mitosis, has been shown to be the best prognostic factor in LN^-^ patients younger than 55 years [13, 14]. Proliferation markers make up an important part of prognostic gene signature markers [12], and according to the latest guidelines of the Norwegian Breast Cancer group (NBCG), high proliferation is an indicator for chemotherapy [15]. However, even though proliferation markers are statistically very prognostic they are still not specific enough, as only 30–40% of patients with highly proliferating tumors will develop distant metastases [13]. As such, there is a need for markers highlighting additional aspects of tumor cell aggressiveness.

The metastatic potential of tumor cells depends on invasion through the extracellular matrix (ECM). This invasion is a multistep process involving cellular deformation and degradation of the ECM [16]. The myristoylated alanine-rich C-kinase substrate (MARCKS) family of proteins function in cytoskeletal regulation, protein kinase C signaling, and calmodulin signaling, and are implicated in cell motility, adhesion, and mitogenesis [17–19]. The MARCKS family of proteins differ in subcellular location and membrane binding affinity, and includes the myristoylated alanine-rich C-kinase substrate-like 1 (MARCKSL1), also known as MARCKS-related protein (MRP) and MARCKS-like protein (MLP) [20]. MARCKSL1 is a membrane-bound actin cytoskeleton regulator [18, 21], associated with tumorigenesis in several cancer types [22, 23]. In breast cancer cell lines, MARCKSL1 knockdown results in decreased migration [24]. Furthermore, MARCKSL1 was the strongest upregulated gene in response to estradiol in estrogen receptor alpha (ERα) positive cells co-cultured with bone cells; suggesting a more aggressive tumor phenotype associated with bone metastasis [25]. In contrast, MARCKSL1 also exhibits anti-angiogenic effects in ovarian tumors by suppressing VEGFR2-dependent AKT/PDK-1/mTOR phosphorylation [26]. In addition, in another breast cancer cell line experiment, MARCKSL1 was shown to suppresses LOXL2 induced oncogenesis and stimulating apoptosis [27], thereby acting as a tumor suppressor. The role of MARCKSL1 in tumor progression thus remains to be elucidated.

In a previous study by the authors, MARCKSL1 protein expression was found to be the strongest prognosticator for metastasis-free survival in node-negative breast cancer patients, with additional value in those with high proliferation [28]. Patients with high MARCKSL1 protein expression showed a 44% survival at 15 years follow up, versus 92% survival in those with low expression, yielding a hazard ratio (HR) of 5.1, confidence interval (CI) 2.7–9.8. Since then, few other studies on the MARCKS family of proteins in breast cancer have been published, and the results are conflicting. This calls for further studies on MARKSL1, examining its validity as a clinical biomarker.

Therefore, in the current study we aim to validate the prognostic value of MARCKSL1 protein expression in a new cohort of LN^-^ breast cancer patients. The MARCKSL1 expression was compared with classical prognosticators such as age, tumor diameter, grade, hormone receptor status, presence of tumor-infiltrating lymphocytes (TILs) and proliferation, with distant metastases free survival as the endpoint.

## Materials and methods

### Patients and pathology

**Fig 1:**
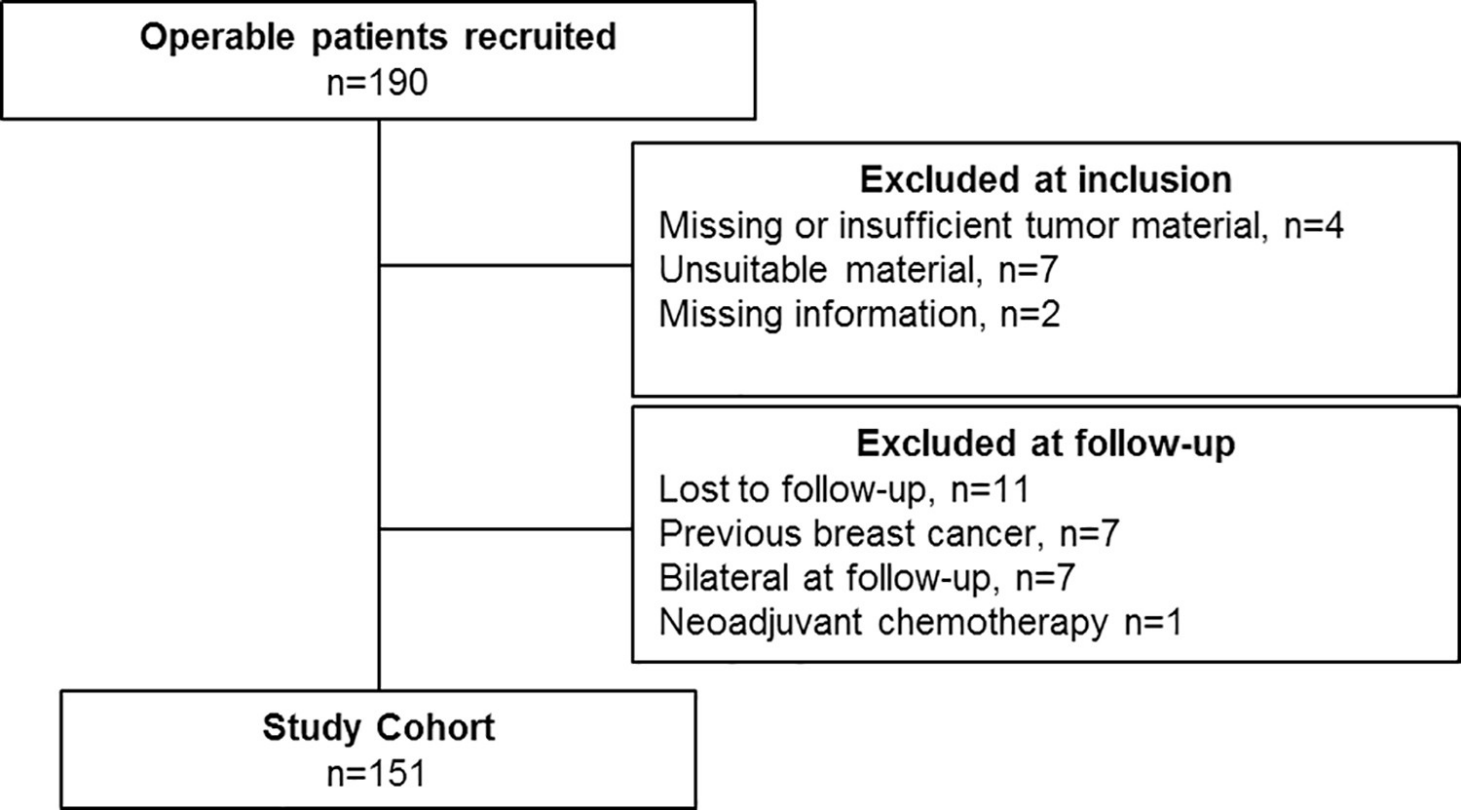
REMARK diagram illustrating patient flow in the study.

Prior to commencement, the study was approved by the Norwegian National Research Ethics Committees/Regional Committees for Medical and Health Research Ethics (REC) West (REC number 210.04). The REMARK guidelines for reporting tumor marker studies were followed [29]. All 190 patients, <71 years of age at diagnosis, were diagnosed with invasive, operable (T_1,2_N_0_M_0_) breast cancer at the Stavanger University Hospital (SUS), between January 15, 2002 and December 22, 2004. Thirteen patients could not be assessed for MARCKSL1 expression, and 26 patients were lost to follow up, had contralateral breast cancer either prior to inclusion or at follow up, or had received neoadjuvant chemotherapy, leaving 151 patients for analysis (Fig 1). There were no significant differences between the original 190 and final 151 cases in any of the features analyzed. The patients were treated according to the national guidelines of the Norwegian Breast Cancer Group. The tumor size was measured in the fresh specimens following excision and cut in slices of 0.5 cm. The axillary fat was examined macroscopically and all detectable lymph nodes were prepared for histology. The median number of identified lymph nodes was two (range 1–21). All tissues were fixed in buffered 4% formaldehyde and embedded in paraffin. Histological sections (4 μm) were made and stained with hematoxylin–erythrosine–saffron (HES). Histological type and grade were assessed by two pathologists (EG and JPAB) according to the World Health Organization criteria [30]. MAI was assessed as described elsewhere [31].

### Immunohistochemistry

ER and progesterone receptor (PR), PPH3, Ki-67, cytokeratin 5/6 (CK5/6), human epidermal growth factor receptor 2 (HER2), and MARCKSL1 expression were determined by immunohistochemistry (IHC) in whole sections. Antigen retrieval and IHC techniques were based on DAKO technology [9]. In brief, formalin fixed paraffin-embedded (FFPE) sections, 4 μm thick, serially sectioned following HES sections, were mounted onto silanized slides (#S3002, DAKO, Glostrup, Denmark). Antigen retrieval was performed with a highly stabilized retrieval system (ImmunoPrep; Instrumec, Oslo, Norway) using 10 mM Tris/1 mM EDTA (pH 9.0) as the retrieval buffer. Sections were heated for 3 min at 110°C followed by 10 min at 95°C then cooled to 20°C. ER (clone SP1, Neomarkers/LabVision, Fremont, CA, USA) was used at a dilution 1:400. PR (clone SP2, Neomarkers/LabVision) was used at a dilution of 1:1000. Rabbit polyclonal anti-PPH3 (ser 10) (Upstate #06–570; Lake Placid, NY) was used at a dilution of 1:1500. Ki-67 (clone MIB-1, DAKO, Glostrup, Denmark) was used at dilution 1:100. CK 5/6 (Clone D5/16 B4, Dako, Glostrup, Denmark) was used at a dilution of 1:100. Mouse monoclonal MARCKSL1 (Clone K53, sc-130471, Santa Cruz Biotechnology, Santa Cruz, CA, USA) was used at a dilution of 1:300. All antibodies were incubated for 30 min at 22°C. The EnVision^TM^ FLEX detection system (Dako, K8000) was used for visualization. Sections were incubated for 5 min with peroxidase-blocking reagent (SM801), 30 min with the primary antibody, 20 min with the EnVision^TM^ FLEX/HRP Detection Reagent (SM802), 10 min with EnVision^TM^ FLEX DAB+ Chromogen (DM827)/EnVision^TM^ FLEX Substrate Buffer (SM803) mix and 5 min with EnVision^TM^ FLEX Hematoxylin (K8008). The slides were dehydrated and mounted. All immunohistochemical stainings were performed using a Dako Autostainer Link 48 instrument and EnVision^TM^ FLEX Wash Buffer (DM831). For HER2 assessments, DAKO HercepTest^TM^ was used according to the procedures of the manufacturers.

### Quantification of PPH3, Ki67, CK5/6, ER, PR, HER2, TILs and MARCKSL1

The PPH3 expression was evaluated by subjective count by counting the number of PPH3-positive objects at 40× by two independent pathologists in 10 adjacent fields of vision (FOV), or a total of 1.59 mm2, in the most PPH3-positive areas. For measuring percentage of Ki-67 positive cells, the semi-automatic interactive computerized QPRODIT system (Leica, Cambridge) was used [32]. For each measurement 250–350 fields of vision were selected, the Ki-67 percentage was defined as [(Ki-67 positive)/ (Ki-67 positive + Ki-67 negative)] x 100. The percentage of CK5/6 positive tumor cells in each tumor was scored using a continuous scale of 0–100%. In the final analysis, all tumors with any CK5/6 staining in tumor cells were grouped as being positive as described before [9]. ER was scored as positive when nuclear staining was present in >1% and scored negative when <1%. PR was scored as positive when nuclear staining was present in >10%, borderline 1–10% and negative when <1%. HER2 was scored according to the DAKO HercepTest scoring protocol. All 2+ and 3+ cases were regarded as positive. All sections were independently scored by two of the authors (BH and EJ). Tumor infiltrating lymphocytes (TILs) were scored semi-quantitatively in HE-stained tissue sections according to the presence or absence of stromal TILs. The relative amount of TILs in the tumor stroma area was then assessed according to the recommendations described by Salgado et al [33]. The degree of infiltration was scored in the range of 0–100%. Positive TILs were defined as ≥1%.

MARCKSL1 was scored in the same way as in our previous study [28] using the following criteria: overall diffuse cytoplasmic (referred to as cytoplasmic hereafter) staining, membrane staining, and granular staining in 10 high power fields (1.59 mm^2^), usually the invasive front of the tumor (S1 Fig). For each of the criteria scoring from 0 to 3+ (0 = lowest score, 3+ = highest score) was given by assessing both intensity and number of positive tumor cells. For the membrane staining, the Dako HER2 scoring guideline was used. A total MARCKSL1 score was calculated by adding all the scores from the different criteria, resulting in a minimum score of 0 and a maximum score of 9 (S2 Fig). As in Jonsdottir et al, a high MARCKSL1 expression was defined as a score of ≥7 [28]. The slides were scored blinded and separately by two of the authors ER and EJ.

### Survival endpoints

For survival analysis, the main endpoint was distant metastasis-free survival (DMFS). To determine the probability that patients would remain free from distant metastasis, we defined recurrence as any recurrence at a distant site. Patients were censored from the date of their last hospital visit for death from other causes than breast cancer, or local or regional recurrences. If a patient’s status during follow-up indicated a confirmed metastasis without a recurrence date, the last follow-up visit date was used. Age, time to first recurrence and survival time were calculated relative to the primary diagnosis date.

### Statistical analyses

Statistical analysis was performed in SPSS (SPSS, Chicago, IL, USA), version 23. Kaplan-Meier survival curves were constructed and differences between groups were tested by the log-rank test. The relative importance of potential prognostic variables was tested using Cox-proportional hazard analysis (method: Forward, Wald) and expressed as HR with 95% CI. Group wise comparisons were performed using Fisher’s exact test.

## Results

Median age at diagnosis for the included patients was 55 (range 28–70) years with a median follow-up time of 152 months (range 11–189 months). In total, 13/151 patients (8.6%) developed distant metastasis and 11/151 patients (7.3%) died of breast cancer related disease. Survival and tumor related characteristics for distant metastasis-free survival (DMFS) are shown in Table 1.

**Table 1:** DMFS in lymph node-negative breast cancer patients.

Characteristic	Distant metastasis			
	Event / at risk (%)	Log rank p value	HR	95% CI
**Age**				
<55	6/77 (92)	0.466	1.5	0.5–4.5
≥ 55	7/74 (91)			
**Tumor diameter**				
<2	5/121 (96)	<0.001	9.3	2.8–31.0
≥2	8/30 (73)			
**Nottingham grade**				
1	2/24 (92)	0.451		
2	4/60 (93)		1.0	0.2–5.2
3	5/34 (85)		2.3	0.4–12.3
**Estrogen receptor**				
Negative	3/24 (88)	0.413	1.7	0.5–6.2
Positive (≥ 1%)	10/126 (92)			
**Progesterone receptor**				
Negative	3/38 (92)	0.221	1.1	0.3–4.3
Positive (≥ 10%)	8/108 (93)		5.3	0.7–42.6
Borderline (1–9%)	1/3 (67%)			
**Her2**				
Negative	6/27 (78)	1.000	1.0	0.3–3.6
Positive	4/19 (79)			
**Triple negative**				
Any receptor positive	11/137 (92)	0.062	3.8	0.8–17.2
Triple negative	2/7 (71)			
**MAI**				
<10	8/108 (93)	0.296	1.8	0.6–5.5
≥ 10	5/40 (88)			
**MAI**				
0–2	4/66 (94)	0.473		
3–9	4/42 (91)		1.6	0.4–6.5
≥10	5/40 (88)		2.2	0.6–8.3
**Ki-67**				
0–9%	5/69 (93)	0.477	1.5	0.5–4.6
10–100%	8/78 (90)			
**Ki-67**			1.7	
<15%	5/81 (94)	0.324		
15–30%	4/39 (90)			0.5–6.5
>30%	4/27 (85)		2.5	0.7–9.9
**PPH3**				
<13	7/94 (93)	0.427	1.6	0.5–4.6
≥13	6/54 (89)			
**TILS**				
0%	3/72 (96)	0.063	3.2	0.9–11.6
≥1%	10/77 (87)			
**CK5/6**				
Negative	0/11 (100)	0.324	-	-
Positive (≥1%)	13/138 (91)			
**MARCKSL1 total score**				
Low (0–6)	13/146 (91)	0.498	-	-
High (7–9)	0/5 (100)			
**MARCKSL1 total score in patients with PPH3>13**				
Low (0–6)	6/49 (87)	0.417	-	-
High (7–9)	0/5 (100)			

CI, confidence interval; HR, hazard ratio; MAI, mitotic activity index

**Fig 2:**
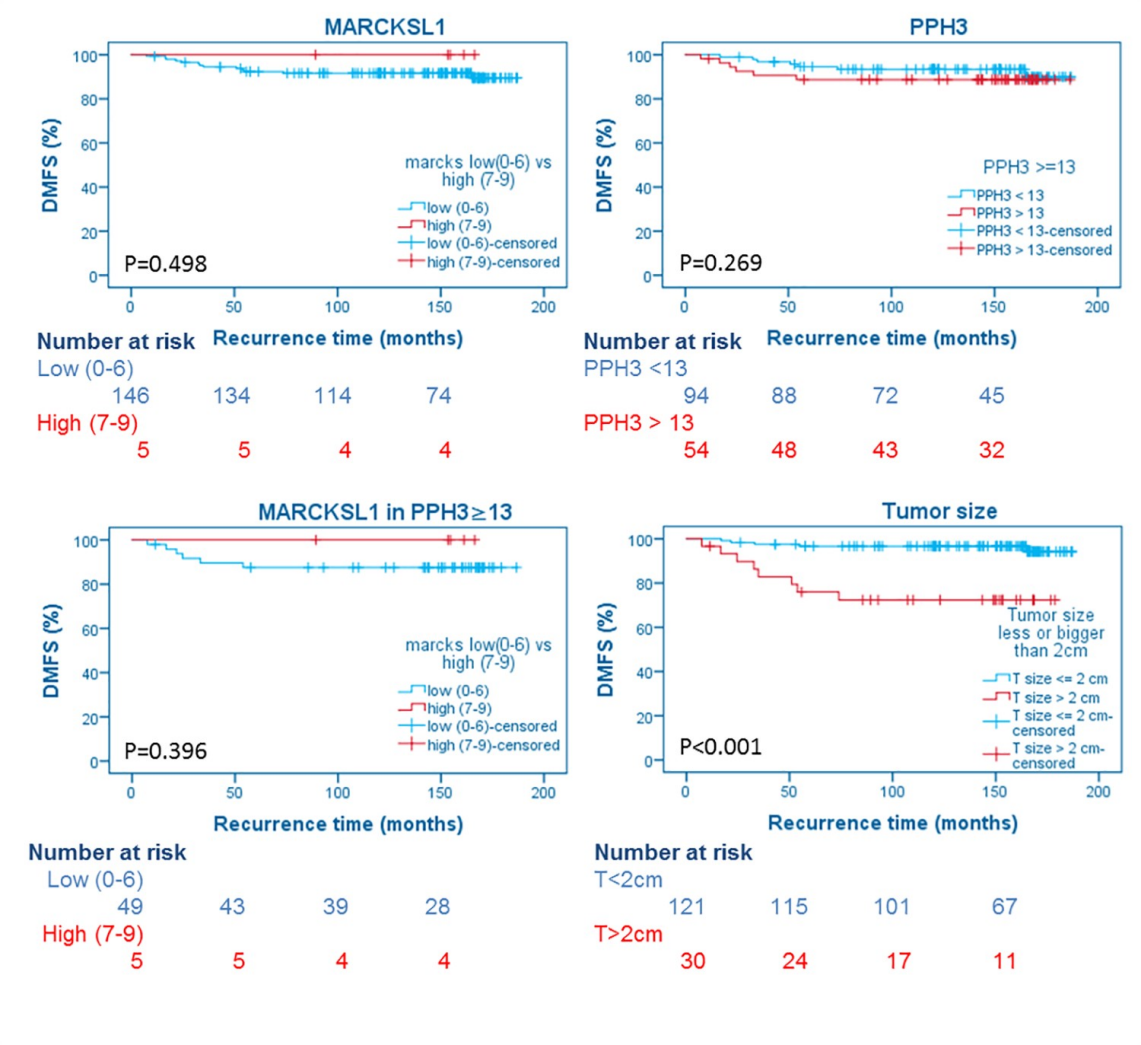
Long-term recurrence-free survival curves according to PPH3 status, MARCKSL1 protein expression score, MARCKSL1 protein expression score in patients with PPH3 ≥ 13, and tumor size. (DMFS, distant metastasis-free survival).

Of the analyzed factors, only tumor diameter showed prognostic value (HR = 9.3, p<0.001, 95% CI = 2.8–31.0). MARCKSL1 protein expression was not a significant prognostic factor (p = 0.498). MARCKSL1 membrane score (p = 0.263), cytoplasmic score (p = 0.221) and granular score (p = 0.307) were not independently prognostic for DMFS either. Of the 151 patients, five (3%) had high MARCKSL1 protein expression and four (3%) had no MARCKSL1 expression in the cytoplasm, membrane or granules. MARCKSL1 protein expression did not have additional prognostic information in the group of patients with PPH3 ≥13 (Fig 2). No patients with low proliferation (MAI<10) showed high MARCKSL1 protein expression, therefore this subgroup could not be assessed for additional prognostic information by MARCKSL1, as suggested previously [28].

In the multivariate analyses, we included all variables showing p <0.1 with regards to DMFS in univariate analysis (tumor size, triple negative receptor status, TILs ≥1%). Tumor size was the strongest prognostic factor for DMFS (n = 140, 93%), and the only significant factor.

MARCKSL1 protein expression was higher in ERα and PR negative tumors (p = 0.029 and p = 0.012, respectively), and tumors with high proliferation (MAI ≥10 (p = 0.004) or PPH3 ≥13 (p = 0.005)), but did not differ between categories of age ≥55, Nottingham grade, tumor size (≥2 cm), HER2, triple negative receptor status, Ki-67 ≥15 or ≥30. Tumor size was not a significant prognostic factor in ERα+ patients <55 years (Kaplan Meier p = 0.286), and no other factors were significant in this group. There was no difference in survival between Luminal A (ERα+, Ki-67 <15%) and Luminal B (ERα+, Ki-67 ≥15%) (Kaplan Meier p = 0.362) patients.

**Fig 3:**
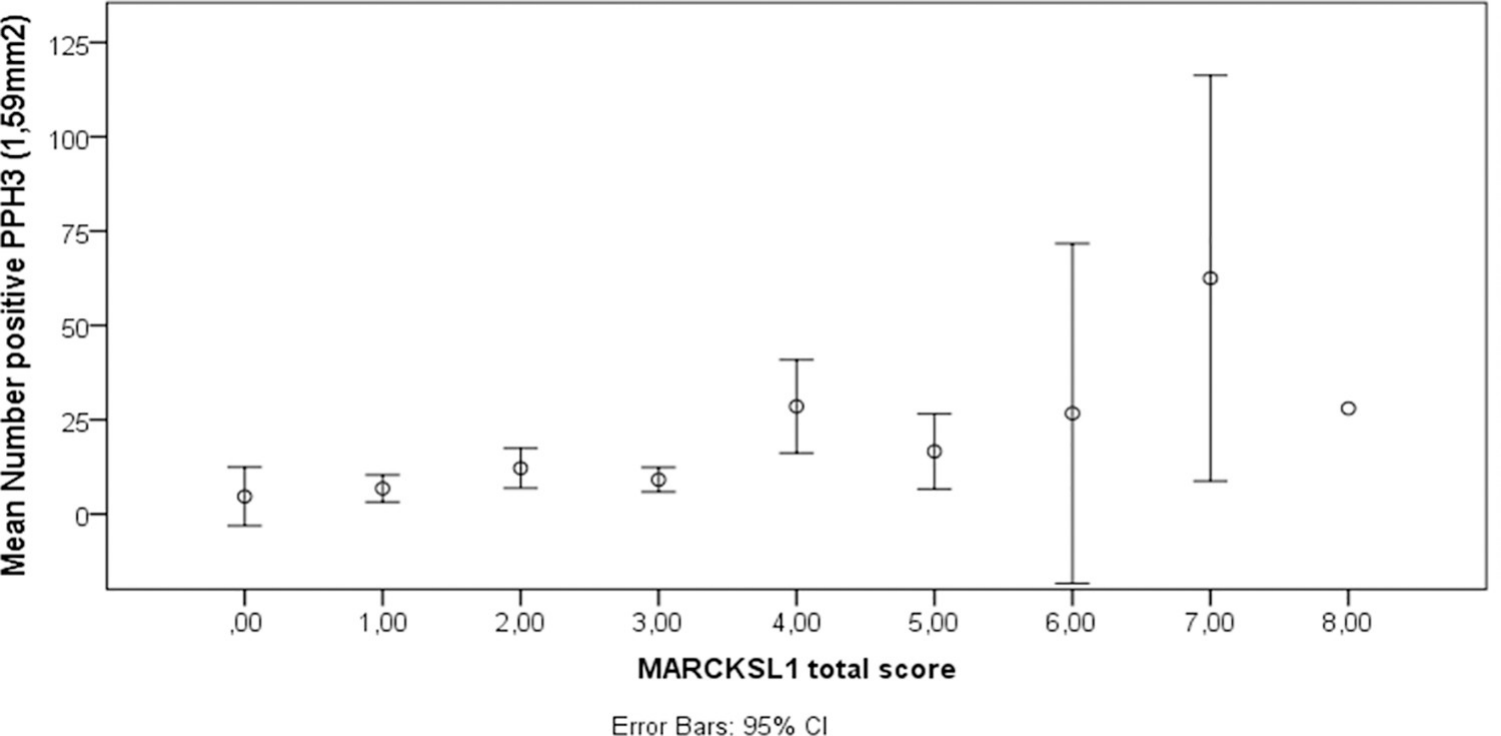
Correlation between MARCKSL1 total score and PPH3 in breast tumors.

MARCKSL1 protein expression was significantly correlated to tumor size (Spearman’s rho 0.220, p = 0.007), Nottingham grade (rho 0.263, p = 0.002), MAI (rho 0.316, p <0.001), PPH3 (rho 0.317, p <0.001) (Fig 3) and Ki-67 (rho = 0.269, p = 0.001).

Finally, we compared our validation cohort to the previous cohort to assess possible causes of the lack of prognostic value of MARCKSL1 (S1 Table). The validation cohort had fewer tumors larger than 2 cm (20% vs. 30%, p = 0.018), younger patients at diagnosis (51% versus 40% <55 years, p = 0.035), higher frequency of chemotherapy (56% vs. 15%, p <0.001) and endocrine therapy (24% vs 6%, p <0.001). At follow-up in the new cohort, there were fewer distant metastases (9% vs 15%, Fisher’s exact test p = 0.055) and fewer deaths from breast cancer (7% vs 11%, Fisher’s exact test p = 0.312) although not a significant reduction in either. There were more PR positive and fewer HER2 positive tumors in the validation cohort. In contrast with the validation study, high MARCKSL1 total score was associated with ER positivity and lower age in the previous cohort, whereas in the new cohort it was associated with ER negativity and not with age.

## Discussion

This study was performed as an independent validation of the prognostic value of MARCKSL1 protein expression in LN^-^ breast cancer patients under 71 years of age. To ensure correct diagnostics and optimal treatment options for patients, it is vital to validate suggested prognostic factors in new cohorts. Our study represents an awaited validation study for the use of MARCKSL1 as a prognostic marker in node-negative breast cancer. In the study by Jonsdottir et al, all LN^-^ patients diagnosed between 1993 and 1998 at the Stavanger University Hospital (SUH) were included [28]. For our validation study, we have collected samples from the same population as the exploratory cohort, and all available consecutive LN^-^ breast cancer patients <71 years at SUH in the study period (2002–2004) were included.

The current study showed that MARCKSL1 protein expression was not a significant predictor for recurrence in the validation cohort (p = 0.498). Additionally, previously validated prognostic factors (Ki-67, PPH3, and MAI) were not prognostic either, leaving only tumor size (with a cut-off at 2 cm) as a significant predictor for recurrence. Only five tumors (3%) were scored as high MARCKSL1 protein expression in our validation cohort, compared to 8% in the discovery cohort. None of the five experienced any metastasis. MARCKSL1 correlated significantly to tumor proliferation as measured by Ki-67, MAI and PPH3. High tumor proliferation, measured by, for example Ki-67, is an indicator for chemotherapy according to the NBCG guidelines [15]. As a result, chemotherapy reduces these patients’ risk of recurrence but also negates the prognostic appearance of Ki-67. We speculate that while MARCKSL1 was prognostic in an earlier population, changes in chemotherapy guidelines have altered the overall survival and the specific need for prognostic factors in Norwegian breast cancer patients [1, 34].

Few other studies have investigated the role of the MARCKS family of proteins in breast cancer. In a previous study by the co-authors [28], increased MARCKSL1 gene expression was not found to be predictive; rather, low MARCKSL1 mRNA levels were predictive of recurrence. Although we did not perform gene expression analysis in the validation cohort, this discrepancy may be due to different activity of MARCKSL1 dependent on its phosphorylation status. Phosphomimetic MARCKSL1 has been shown to inhibit migration, whereas dephosphorylated MARCKSL1 induces migration in neurons [19]. Differential phosphorylation could explain the apparent oncogenic and antitumor effects of MARCKSL1 reported in different studies. The IHC analyses performed here do not discriminate between protein phosphorylation statuses.

Alternatively, MARCKSL1 activity may be suppressed by microRNA suppression of protein translation. In fact, in a human breast carcinoma cell line, knockdown of MARCKSL1 by 5’isomiR-140-3p overexpression led to a decrease in the migratory potential of cells [24], in line with the findings of Jonsdottir *et al*. [28].

Reasons for the lower MARCKSL1 scores overall in the current compared to previous cohort could be that the patients in the validation cohort are diagnosed at an earlier stage, with younger patients and smaller tumors (S1 Table). Additionally, the change in chemotherapy type and frequency may contribute to fewer recurrences and deaths (9% vs 15% distant metastases in the current vs previous cohort, respectively). A key explanation is the introduction of mammography screening and the change in chemotherapy indications and type in recent years, resulting in earlier stages and younger age at diagnosis and fewer recurrences and cancer deaths [34]. The population of women diagnosed with breast cancer changes over time, with younger and earlier staged patients increasing after the gradual introduction of the national screening program in Norway from 1996 [2, 15]. Concurrently, the chemotherapy regimen was changed from cyclophosphamide, methotrexate and fluorouracil (CMF) to the more effective anthracycline-based fluorouracil, epirubicin and cyclophosphamide (FEC) [35]. In addition, the indication for endocrine therapy (in addition to HR+) changed from tumor size > 20mm in 2000, to a tumor size >11mm in 2003 [2, 3, 35]. Increased use of endocrine therapy may also contribute to the increased metastasis-free and overall survival in the validation cohort compared to the exploratory cohort [2]. Furthermore, breast cancer recurrence is known to occur up to 20 years following diagnosis [4], which extends beyond the time span of both this and the previous study.

Possible limitations to the current study include the relatively small study size, lack of mRNA measurements, and the change in treatment regimes. Due to increased survival in recent years, a greater study size may be needed to obtain sufficient numbers of recurrences. Additionally, had mRNA measurements been performed, these could explain whether reduced MARCKSL1 expression was due to reduced gene expression or other factors. Finally, as mentioned, other studies have observed opposite effects of MARCKSL1 depending on its phosphorylation status [19]. This is also the case with the much more studied MARCKS [36].

In conclusion, in this second cohort MARCKSL1 protein expression could not be confirmed as a prognostic factor. Thus, with changes both in the diagnosed population and how they are treated, the search for prognostic biomarkers must continue in new directions. Further studies are needed to reveal the potential biological role of this protein in breast cancer.

## Supporting information

S1 Fig Example of MARCKSL1 (myristoylated alanine-rich C kinase substrate like-1) staining (brown staining). A) Strong membrane staining. B) Strong granular staining. C) Strong cytoplasmic staining. D) Negative/Weak staining.
(PDF)

S2 Fig **MARCKSL1 expression scores** in a) validation cohort and b) discovery cohort (Jonsdottir et al. 2012).
(PDF)

S1 Table Comparison of the discovery cohort and the validation cohort. (PDF)

S2 Table Patient characteristics data. (XLSX)
