# Supplementary material for: Validation study of MARCKSL1 as a prognostic factor in lymph node-negative breast cancer patients
Source: PLoS One. 2019 Mar 11;14(3):e0212527. doi: 10.1371/journal.pone.0212527 (PMC6411117; doi:10.1371/journal.pone.0212527)
Supplement: S1 Fig — A) Strong membrane staining. B) Strong granular staining. C) Strong cytoplasmic staining. D) Negative/Weak staining. (PDF) [file pone.0212527.s001.pdf]

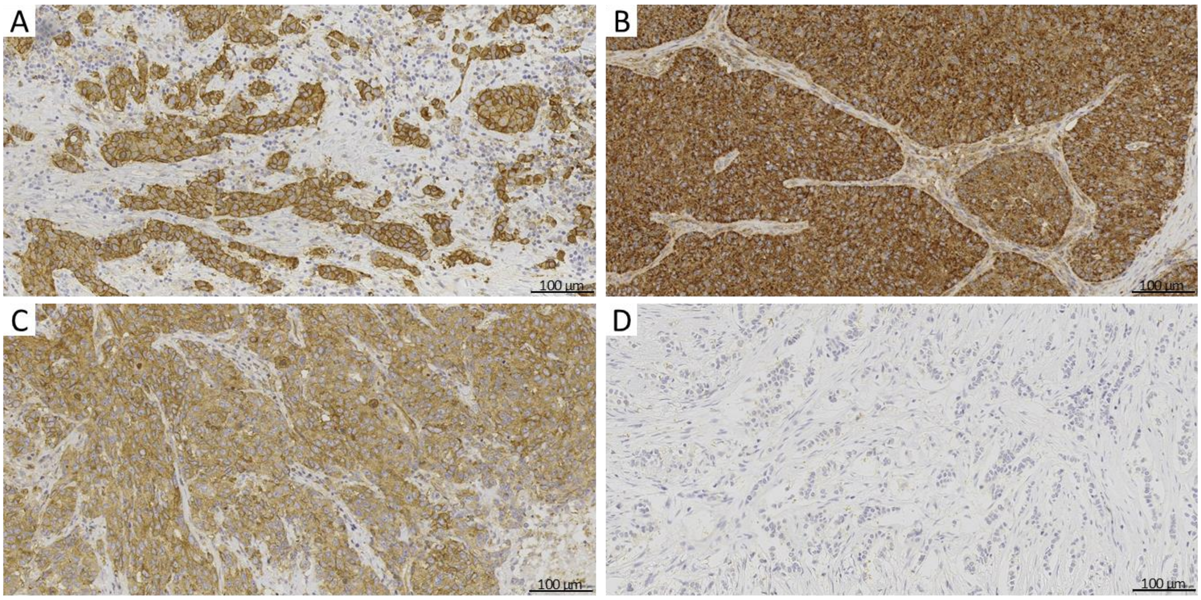

S1 Fig. Example of MARCKSL1 (myristoylated alanine-rich C kinase substrate like-1) staining (brown staining). A) Strong membrane staining. B) Strong granular staining. C) Strong cytoplasmic staining. D) Negative/Weak staining.
