# Supplementary figures and images for: Validation study of MARCKSL1 as a prognostic factor in lymph node-negative breast cancer patients
Source: PLoS One. 2019 Mar 11;14(3):e0212527. doi: 10.1371/journal.pone.0212527 (PMC6411117; doi:10.1371/journal.pone.0212527)

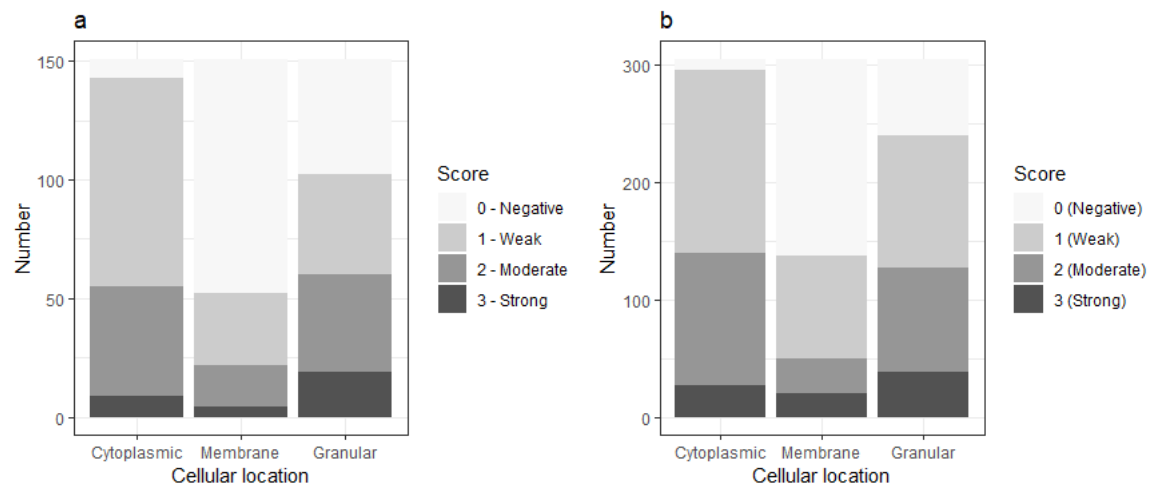

**S2 Fig. MARCKSL1 expression scores in a) validation cohort and b) discovery cohort (Jonsdottir et al. 2012)**

Supplement: S2 Fig — MARCKSL1 expression scores in a) validation cohort and b) discovery cohort (Jonsdottir et al. 2012). (PDF) [file pone.0212527.s002.pdf]
