# Supplementary material for: Validation study of MARCKSL1 as a prognostic factor in lymph node-negative breast cancer patients
Source: PLoS One. 2019 Mar 11;14(3):e0212527. doi: 10.1371/journal.pone.0212527 (PMC6411117; doi:10.1371/journal.pone.0212527)
Supplement: S1 Table — (PDF) [file pone.0212527.s003.pdf]

Characteristic			
	Jonsdottir et al. 2012 (n=305)	Validation (n=151)	P (Fisher's exact test)
**Cohort**	**1993-1998**	**2002-2004**	
**Age**			0.035
<55	40%	51%	
≥ 55	60%	49%	
**Tumor diameter**			0.018
<2	70%	80%	
≥2	30%	20%	
**Nottingham grade**			0.158
1	30%	20%	
2	44%	51%	
3	26%	29%	
**Estrogen receptor**			0.327
Negative	17%	16%	
Positive (≥ 10%)	81%	84%	
Borderline (1-9%)	2%	0%	
**Progesterone receptor**			<0.001
Negative	22%	26%	
Positive (≥ 10%)	62%	72%	
Borderline (1-9%)	16%	2%	
**Her2+**			<0.001
Negative	87%	60% [a]	
Positive	13%	40% [a]	
**Triple negative**			0.648
Negative	87%	84% [a]	
Positive	13%	16% [a]	
**MAI**			0.363
<10	71%	66%	
≥ 10	29%	34%	
**Ki-67**			0.167
0-9%	52%	44%	
10-100%	48%	56%	
**PPH3**			0.676
<13	61%	64%	
≥13	39%	36%	
**CK5/6**			0.845
<10	93%	93%	
≥10	7%	7%	
**TILs**			<0.001
<1%	13%	48%	
≥1%	87%	52%	
**MARCKSL1 total score**			0.097
Low (0-6)	92%	97%	
High (7-9)	8%	3%	
**Chemotherapy**			<0.001
Yes	15%	55%	
No	85%	45%	
**Endocrine therapy**			
Yes	6%	23%	<0.001
No	94%	77%	
**Distant metastasis**	15%	9%	0.055
**Deaths from breast cancer**	11%	7%	0.312

[a]HER2 status missing for 105 patients
